# Removal of the Placenta in the Early Months of Pregnancy by
Evulsion

**Published:** 1859-04

**Authors:** 


### ECLECTIC DEPARTMENT,

### AND SPIRIT OF THE MEDICAL PERIODICAL PRESS.

---

### *Removal of the Placenta in the Early Months of Pregnancy by Evulsion.*

By O. C. Gibbs, M. D., Frewsburg, Chautauque Co., N. Y.

In the discussion of this subject before the New York Academy of Medicine, as reported for, and published in the October No. of the Monthly, several obstetricians expressed the opinion that instrumental delivery of the placenta after abortion, even in alarming cases of hæmorrhage, was unsafe and unnecessary.

To take part in a discussion, in which men of such extensive experience and wide-spread reputation have participated—men whose opinions are so worthy of respect and confidence, as are Drs. Barker, Gardner, Hubbard, Sewall, Underhill, &c., may seem like the height of presumption on our part. But, holding to some opinions of a positive character upon this subject, we trust we shall be exempted from the charge of egotism in giving them expression, though our field of observation may be more limited than that of another man who may hold opposite opinions.

We would preface our remarks, by saying that our opinions do not differ materially from those of Dr. A. K. Gardner, as at different times expressed. We might differ in the choice of instruments; also, perhaps, as to the time for their use. But more of this further on.

Uterine hæmorrhage, if considerable, is always a source of anxiety, danger and alarm, and never more so than when it succeeds delivery, whether mature or premature. In such cases, it retards recovery, endangers life, and is often the immediate cause of death.

This last statement, we presume, will not be denied, for it is too well known that deaths from hæmorrhage, after labor or abortion, are, by no means, infrequent, and when death is not the direct result, from loss of blood, the patient is frequently so prostrated as to be many months in recovering perfectly, and often, other diseases are superinduced, leading more remotely to the grave. Dr. Thomas F. Cock, of New York, says, "such hæmorrhages are of importance, because, in many cases, they have proved fatal; and their treatment demands some peculiarities beyond that usually pursued in flooding." (See New York Journal of Medicine, July, 1857.) Hence the importance of fully understanding the causes of such hæmorrhages and their most appropriate methods of treatment.

Dr. Gardner has truly said, "there is no time for vacillation in opinion or action; no time to run home to hunt up some method of treatment. The surgeon, if he finds himself at at fault in this anatomy, while an assistant compresses the artery, may turn to his manual for light respecting the course of an artery; but in the floodings of the parturient there can be no temporizing. (See American Medical Monthly, vol. iii., p. 420.)

It is not infrequently the case, that in the early months of pregnancy the uterus expels the fœtus, while the placenta is retained, but partially detached from the uterine walls, in which event, excessive hæmorrhage is an accident of difficult prevention. In all such cases as have fallen under our observation, the hæmorrhage has immediately ceased on the complete detachment and removal of the placenta. The question now under consideration is whether hæmorrhage, under such circumstances, shall be arrested beyond the probable contingency of a return, *by an immediate removal of the placenta with instrumental aid, if need be,* or whether the case shall be trusted to those uncertain remedies, styptics and the tampon, aided by time and the recumbent posture? In our ten years of rather limited comparative experience, in cases of alarming uterine hæmorrhages following abortion, we have lost no time in detaching and removing the placenta, and we have seen no reason to regret our course.

Even in cases of hæmorrhage before the uterus has expelled the ovum, *where the life of the patient is in jeopardy, and all hopes of saving the life of the embryo have vanished,* we have not hesitated to separate the embryo from all vascular connection with the uterus, and remove the products of conception; in which event the hæmorrhage has invariably ceased. The propriety of this latter course is not at present under consideration; we have simply to deal with a partially detached and retained placenta. In such cases how shall the removal be accomplished? We answer, *with the fingers if it can be, with instruments if we must.* What are the objections to such removal? No one hesitates to introduce the hand into the womb to accomplish turning, or to remove an attached placenta at full term. The irritation produced by the introduction of a suitable instrument to remove a retained placenta following abortion in the earlier months, is much less than that caused by the introduction of the hand at full term, and infinitely less than that caused by the disintegration and decay of the retained placenta. For the last eighteen months, the instrument which we have used is that described and figured by Dr. Carey, of Dayton, Ohio, in the American Medical Monthly, vol. vii., p. 4, and subsequently in the Western Lancet, vol. xviii., p. 276. Though designed for the removal of the ovum, in cases of necessity, it is decidedly the best and safest instrument we ever used for the removal of the secundines. We certainly prefer this instrument to the "crotchet" of Dr. Dewees, or the "placental forceps" of Dr. Henry Bond. In regard to the propriety of the removal of the secundines after abortion, we might cite various high authorities, but we will content ourselves simply with quoting Dr. Churchill. He says, "if the fœtus alone be expelled, we may wait a while, (if no flooding occurs,) to see if the uterine efforts will not detach the secundines; if not, perhaps we may be able to reach the lower portion of them with the finger, and gradually withdraw them; if this fail, we may frequently succeed with the ergot of rye. But there are many cases in which none of these plans will succeed. Are we then to leave the case to nature? We know that after a time, the shell of the ovum will putrefy, dissolve, and be discharged; but experience too often proves that this process involves considerable danger; danger of hæmorrhage first, and afterwards of uterine phlebitis. With regard to the danger of uterine phlebitis from absorption of a putrid ovum, it is sufficiently imminent to warrant interference if we are called early enough." Dr. Churchill does not recommend, but rather reprobates the use of instruments for the removal of the secundines, when the same benefits can be obtained by the fingers, as perhaps first recommended by Dr. Wainwright. Upon what grounds this reprobation is made we cannot conceive, for the judicious use of a proper instrument will produce as little, if not less disturbance, as the necessary manipulations of the fingers within the uterus. We are tempted to quote here a remark by Dr. A. K. Gardner, in an able paper upon uterine hæmorrhage: "How shall the small and fragile placenta be seized hold of and withdrawn? Some have recommended the introduction of one finger into the uterus, and bringing down one edge of the placenta, and twisting it round and round, not only thus to detach the entire mass, but to also so shape it that it may the more easily pass through the os. Where this can be done, by all means do so! But it should be remembered, that in the great mass of cases it is impossible to reach the os, so as to pass one finger into the cavity; far less to effect any good result, if it arrive there, to say nothing of the utter impossibility of aiding the finger with the thumb of the same hand.'' (See American Medical Monthly, vol. iii., p. 441.)

We propose to give two cases; the first and last in which we have used Dr. Carey's instrument, which will fully illustrate the time and the circumstances when and in which we seek instrumental aid. The first case has been previously reported, but it none the less subserves our present purpose.

Case I. June 18th, 1857, we were called to see Mrs. M., aged 40 years. We found her sitting up, and she said she was feeling very well. A few hours before, she had miscarried, at three and a half months, with but trifling pain and no hæmorrhage. She had previously borne several children, the youngest of which was now ten years of age. The placenta had not come away, and it was on that account that she sought medical aid. On examining the case per vaginam, we traced the slender cord within the uterus. Mrs. M. was a very fleshy woman, of firm muscle, and the soft parts not having been distended by the passage of a full-grown child, resisted the introduction of the hand into the vagina. Pressing the hand firmly up, we could just introduce the end of the finger within the os uteri, but not sufficient to reach the placenta, or with the finger to depress the uterus. In our efforts at extraction, the slender placental cord was broken, after which the womb seemed to float before the finger, and all further efforts at extraction, with unaided fingers, were utterly useless. There was no hæmorrhage, and consequently no necessity for immediate action, but because of the extreme anxiety, on the part of the patient, to have the placenta removed immediately, and because our business would call us daily, for a few days, some ten or twelve miles away, were the above mentioned efforts made at extraction. We now gave ergot in full and repeated doses, utterly without effect. We now left our case as we found it, instructing the friends to lose no time in sending for us should hæmorrhage occur.

We had no idea of abandoning the case wholly to nature. Dr. Huston, of Philadelphia, says, "time, rest and opium, are the grand remedies in abortion, for which there are no substitutes." We were disposed to give the patient the benefit of all these, while we took a little "time" ourselves to prepare for an emergency, which we had every reason to expect would soon come. We instructed our friend Fox, an ingenious gunsmith, to make an instrument, immediately after Dr. Carey's model, which instrument was held in reserve for future use, when the urgency of the symptoms should demand active interference. During the night of the third day, we were summoned, in great haste, to the bed-side of our patient, who was reported as flowing profusely. Residing two miles away, of course some little time elapsed from the commencement of flowing to the time of our arrival. We went to the case with considerable misgiving; patient and persevering efforts for extracting the secundines had been fruitlessly made with unaided hands; no better result could be expected from a repetition of the effort, and we had yet to learn what confidence could be placed in the instrument which we were now prepared to bring to our aid. It was true, we could resort to the tampon, but this would only protract the case; it would not remove the placenta, nor insure against a recurrence of the hæmorrhage on its removal. We found our patient almost senseless and pulseless from loss of blood, and friends were weeping in an agony of grief at the prospects of her immediate dissolution. A dose of camphor and brandy was administered immediately, and we sat down to our patient without delay. The vagina was emptied of clots, and the hand was pressed firmly up, until the end of the finger reached the os uteri. Carey's instrument was now passed up, guided by the finger, until the claw entered the os; it was now turned to the one side, with the intention of bringing its convexity in immediate contact with the uterine walls, and then gently carried up to the fundis. From this position the instrument was made to sweep the entire cavity of the uterus, carefully keeping the polished surface of the convexity of the claw facing towards its walls. The instrument was now turned on its long axis, and carefully brought down; the placenta was immediately felt in contact with the end of the finger, which was still kept in the os; with the finger acting as one blade of a pair of forceps, and the claw of the instrument as the other, the placenta was grasped, and at once removed. The hæmorrhage immediately ceased, and the patient made a good recovery. Though the time was not accurately noted, it is presumed the placenta was removed in less than two minutes from the time of sitting down to the patient. (See a former paper of ours in the Western Lancet, vol. xviii., p. 645.)

Case II. October 10th, 1858, was called to see Mrs. B., a feeble woman of nervous temperament. She was deadly pale, and almost pulseless from loss of blood. She had been flowing more or less profusely for the last three days, and had been attended, because of our absence, by Dr. Boyd. Ergot, acetate of lead, opium, gallic acid, and the tampon, had all been used with no permanent effect. The patient was supposed to be about six weeks pregnant, and, from the amount and persistency of the hæmorrhage, all hopes of saving the life of the ovum was abandoned. As soon as these facts were ascertained, we sat down to the patient, emptied the vagina of clots of blood, passed the finger to the os uteri, and then, along the finger, passed Dr. Carey's instrument into the uterus, and with the same manipulation as before described, the ovum was removed. The hæmorrhage immediately ceased, and the patient made a good and speedy recovery.

In the discussion before the Academy of Medicine, as reported in the October No. of the Monthly, several gentlemen present deprecated the use of instruments, while they commended the use of the tampon as safe and reliable; all, however, agree in the desirability of immediately removing the secundines, where hæmorrhage is profuse. We are disposed to question both the safety and reliability of the tampon. First, it is not reliable, because it does not remove the cause of the hæmorrhage, which is liable to recur at each removal of the tampon; neither does it perfectly and promptly arrest hæmorrhage, for, however thoroughly the vagina may be plugged, it is still capable of containing more or less blood, and often hemorrhage will continue until the uterus is distended to the size which it had attained previous to the occurrence of abortion. Second, it is not always safe; for, if the patient has flooded until there is imminent danger of immediate death, the cavity of the womb will not infrequently continue to receive blood, after the introduction of the tampon, quite sufficient to greatly enhance the danger, if not to prove actually fatal. It is needless to say that there is always an uncertainty attending the final removal of the tampon, and danger attending its too long use. There is always a liability of uterine inflammation from the presence of the placenta and clot in the uterus, and of phlebitis from the absorption of pus during the process of putrefaction.

Other members present preferred to trust the expulsion of the placenta to the influence of ergot. In the first case above reported, ergot signally failed, and this was not the first time we have seen it do so, when our anxiety was wrought to the highest pitch by the imminence of the danger. Others have been so often disappointed, under like circumstances, as to doubt its influence entirely in promoting uterine contractions.

For ourselves, we do not doubt that a good article of ergot, when properly administered, will increase the force of uterine contractions, in cases of labor, at the full term of uterine gestation. We would, however, say, that our experience justifies the conclusion that there is a difference between *increasing* the force of uterine contractions, when such contractions already exist, at the normal terminus of the gestation period, and in *originating* contractions, when nature is not making normal efforts in the same direction. Dr. Huston says, " the use of the ergot of rye, under these circumstances, is not without inconvenience," for it increases pain and nervous excitement, which are not infrequent and unpleasant attendants in these cases. Dr. Budd objects to the administration of ergot after severe hæmorrhage, " as he has often found it a direct sedative." We presume that others have observed the same sedative action. The following is a case in point. Mrs. R., aged about 35 years, a widow, in very feeble health, has, for several years, been subject to occasional and profuse uterine hæmorrhage. She has received treatment at the hands of several physicians, with no permanent good effect. Four days ago, October 22d, we were called, in the absence of her attending physician, to prescribe. She was flowing profusely, and was very faint and quite exhausted. A diligent search discovered no cause of the hæmorrhage. Suffice it for our present purpose to say, that the tampon was used; cold water was injected into the bowels; acetate of lead and opium, gallic acid and turpentine, were administered by the mouth. The hæmorrhage would return with each removal of the tampon, though, it is true, less actively than at first. Yesterday morning the hæmorrhage began to increase again; the tampon, saturated in a strong solution of alum, was applied. We left two doses of fresh ergot (one drachm each,) with directions that one be taken immediately, the other after an interval of six hours. Nothing else was advised. We were called to the case at nine o'clock last night, and found our patient complaining of universal numbness, and a deathly faintness at the stomach, similar to that which a large dose of opium will produce; yet we were assured that none had been taken. The pulse was reduced from one hundred to sixty, and the surface of the body was covered with a cold, clammy sweat. It is proper to say, there had been no hæmorrhage since morning. From her feelings, the patient was positive internal hæmorrhage was going on. Quinine, with the compound aromatic powder, was administered in repeated doses, and to-day the patient is much improved.

We have reported this case, simply to illustrate the depressing effect of ergot, which, in some cases, might enhance the danger of death.

Dr. Barker says, the tampon in his hands, while it stays the blood for the present, excites uterine contractions, which expel the placenta when the tampon is removed. The tampon will not excite contractions, unless placed within the cervix. It is true that the tampon should always be placed there, but our observations lead us to the conclusion that the vagina is its more general seat.

We fully concur with Dr. Barker, when he says, "there is no question as to the propriety and necessity of at once removing the retained portion of the ovum. The only question is as to the best method of accomplishing this." Upon this last point our opinion has been expressed. The hook of Dr. Dewees should only be used when the placenta is wholly detached, and even then we think a better instrument could be selected. In abortion, when the placenta is wholly detached, we think hæmorrhage is extremely rare; for we have seen hæmorrhage cease on separating the vascular connection between the womb and placenta, even before the latter was expelled. The forceps of Dr. Bond we consider but little better, and, in some hands, might be even more objectionable. The polished and convex surface of Dr. Carey's decidual separator is admirably adapted to glide along the inner surface of the womb, and, with a little management, to separate the placental attachment. We now use it, *without delay*, for the removal of retained secundines, after abortion, *when any considerable hæmorrhage is present;* and we have seen and can see no possible danger from its judicious use. Instead of making it, in such cases, our *dernier*, we make it our first resort. We have little doubt but that the uterus will invariably contract, on the removal of the secundines *in abortion*, yet from habit, we almost invariably give ergot.—*American Medical Monthly.*

